# ENPP1/CD203a-targeting heavy-chain antibody reveals cell-specific expression on human immune cells

**DOI:** 10.1007/s00018-024-05539-y

**Published:** 2024-12-18

**Authors:** Hannah Lorenz, Stephan Menzel, Nataliia Roshchyna, Birte Albrecht, Anna Josephine Gebhardt, Enja Schneider, Friedrich Haag, Björn Rissiek, Ralf Oheim, Friedrich Koch-Nolte, Riekje Winzer, Eva Tolosa

**Affiliations:** 1 Department of Immunology, University Medical Center Hamburg-Eppendorf, Hamburg, Germany; 2 Institute of Innate Immunity, Core Facility Nanobodies, University Hospital Bonn, Bonn, Germany; 3 Department of Neurology, University Medical Center Hamburg-Eppendorf, Hamburg, Germany; 4 Institute of Osteology and Biomechanics, University Medical Center Hamburg-Eppendorf, Hamburg, Germany; 5 German Center for Child and Adolescent Health (DZKJ), Partner Site Hamburg, Hamburg, Deutschland; 6 Department of Diagnostic and Interventional Radiology and Nuclear Medicine, University Medical Center Hamburg-Eppendorf, Hamburg, Deutschland

**Keywords:** ENPP1, CD203a, Nanobody, Human immune cells, GACI, Purinergic enzyme

## Abstract

ENPP1/CD203a is a membrane-bound ectonucleotidase capable of hydrolyzing ATP, cGAMP and other substrates. Its enzymatic activity plays an important role in the balance of extracellular adenine nucleotides and the modulation of purinergic signaling, in soft tissue calcification, and in the regulation of the cGAS/STING pathway. However, a detailed analysis of ENPP1 surface expression on human immune cells has not been performed. Here, we selected VHH domains from human ENPP1-immunized alpacas to generate heavy-chain antibodies targeting ENPP1, and analyzed cell surface expression on all circulating immune cell subsets using flow cytometry. We find high expression of ENPP1 in CD141^high^ conventional dendritic cells (cDC1), while ENPP1 was not detectable on other dendritic cells and monocytes. In the lymphocytic compartment, only CD56^bright^ natural killer cells and mucosal-associated invariant T cells (MAIT) express ENPP1. In contrast, all other T cell subpopulations, CD56^dim^ natural killer cells and B lymphocytes do not or only minimally express ENPP1. In summary, we describe highly cell type-specific expression of ENPP1 in the immune system using a newly generated heavy-chain antibody. This reagent will help to decipher the function of ENPP1 in the regulation of the immune response, allow a quick identification of ENPP1-deficiency and of ENPP1-positive tumors, and constitutes the basis for targeted anti-tumor intervention.

**Supplementary Information:**

The online version contains supplementary material available at 10.1007/s00018-024-05539-y.

## Introduction

ENPP1 (ectonucleotide pyrophosphatase/phosphodiesterase 1, also known as CD203a or PC-1) is a type two transmembrane protein involved in important biological processes such as the regulation of bone and soft tissue mineralization, and the modulation of immune responses. ENPP1 belongs to the ENPP family of ectonucleotidases (consisting of members ENPP1-7), characterized by a conserved phosphodiesterase domain. It usually forms a homodimer on the plasma membrane, but can also be secreted as an enzymatically active monomer [1], and has been found on extracellular vesicles [2]. Through its broad substrate specificity, ENPP1 hydrolyzes adenosine triphosphate (ATP), uridine triphosphate (UTP), cyclic adenosine monophosphate (cAMP), and 2′3′-cyclic guanosine monophosphate-adenosine monophosphate (cGAMP), leading to the production of nucleoside 5′-monophosphates and inorganic pyrophosphate (PP_i_) [3, 4].

ENPP1 was initially identified as a surface protein on murine plasma cells, and defined as a hallmark of plasmacytomas [5, 6]. RNA expression data reveal the presence of *ENPP1* in cartilage, heart, kidney, liver, placenta, and testis, with particularly high expression levels in chondrocytes, osteoblasts and vascular smooth muscle cells [7–9]. Additionally, ENPP1 is overexpressed in ovarian, breast and lung cancer as well as in multiple myeloma [10–13].

ENPP1 degrades ATP to AMP, producing PP_i_. PP_i_ is a potent inhibitor of bone mineralization and ectopic soft tissue calcification. While the degradation of PP_i_ to P_i_ by the tissue non-specific alkaline phosphatase (TNAP) maintains bone mineralization [1, 14, 15], reduced PP_i_ levels, as a consequence of biallelic loss-of-function mutations in *ENPP1,* can result in neonatal vascular calcification (Generalized Arterial Calcification of Infancy type 1, GACI type 1) [16] and a rare form of FGF23-dependent rickets (Autosomal Recessive Hypophosphatemic Rickets type 2, ARHR2). GACI usually occurs during fetal development and can cause neonatal heart failure and arterial hypertension with fatal course within the first six months of life in roughly 50% of the affected individuals. ARHR2 manifests in individuals with and without a history of GACI in early childhood as rachitic skeletal deformities, bone pain and/or short stature. Today, only supportive care with an uncertain risk-benefit ratio is available for GACI and ARHR2, but early diagnosis of rickets and daily treatment with vitamin D and phosphorus can help to reduce deformities in ARHR2 [17]*.* However, enzyme replacement therapy (ERT) for patients with *ENPP1* deficiency is currently tested in a phase 1/2 clinical study (NCT04686175, ClinicalTrials.gov).

ENPP1 also serves as an immune checkpoint in both innate and adaptive immune pathways. During cellular stress or inflammation, elevated ATP levels initiate immune activation through P2 receptors. By degrading ATP to AMP, and the subsequent conversion of AMP to immunosuppressive adenosine by the ectonucleotidase CD73, ENPP1 plays an anti-inflammatory role, contributing to immune homeostasis [3]. In addition, the purinergic enzymes CD38, ENPP1 and ENPP3 can modulate type I interferon response by regulating extracellular cGAMP levels [18–20]. cGAMP is produced by cyclic GMP-AMP synthase (cGAS) in response to cytosolic double-stranded DNA, a danger signal indicative of intracellular pathogens or damaged/cancerous cells, and activates the stimulator of interferon genes (STING). ENPP1 is the dominant extracellular cGAMP hydrolase, and inhibits the cGAS-STING pathway by degrading cGAMP, and therefore downregulates the type I interferon response [20, 21]. By inhibiting this pathway, ENPP1 can promote tumor growth and metastasis, and can therefore be used as a stratification biomarker and target for immunotherapy [22, 23]. By cGAMP degradation in combination with AMP generation, the substrate for the production of immunosuppressive adenosine, ENPP1 expressed on tumor cells enhances tumor growth by reducing both the innate and adaptive anti-cancer responses.

While the function of ENPP1 in the context of cancer is currently under intensive investigation, the cell surface expression on human immune cells is barely known. According to the Human Cell Atlas [24], the gene expression of *ENPP1* in the immune system is low, but with a high cell-type specificity. In addition to conventional antibodies, made up of heavy- and light-chains, camelid heavy-chain only antibodies (hcAbs) (or their variable domains known as nanobodies or VHH) are emerging as powerful tools both for immunostaining and therapeutic use. Originally produced in camelids and cartilaginous fish [25], hcAbs are currently obtained by immunizing llamas or alpacas, or generated artificially from synthetic, non-immune VHH libraries. Due to their small size and elongated CDR3 region, hcAbs may reach epitopes that are not accessible by conventional antibodies [26]. They can be easily fused to Fc domains or generated as bispecific conjugates, resulting in versatile tools that can be used for diagnostic and therapeutic applications.

Here we describe the generation and characterization of anti-human ENPP1 hcAbs, which we used to assess the expression of ENPP1 in human circulating immune cells. We confirmed the highly cell type-specific expression of ENPP1 on CD141^high^ conventional dendritic cells (cDC1), CD56^bright^ natural killer (NK) lymphocytes, and mucosal-associated invariant T (MAIT) cells. In contrast to previous reports, we did not detect ENPP1 expression on circulating human plasmablasts or other B cell populations. Of note, our ENPP1-specific hcAbs did not detect the protein in immune cells of individuals with biallelic *ENPP1* deficiency. By precisely targeting ENPP1 on the cell surface, our hcAbs constitute a valuable tool for the detection of ENPP1 protein expression and may offer the potential to also target ENPP1-expressing tumor cells in vivo.

## Materials and methods

### Origin of samples and isolation of peripheral human mononuclear cells

Buffy coats were obtained from the blood bank of the University Medical Center Hamburg-Eppendorf (UKE). Peripheral blood from healthy volunteers and patients with *ENPP1* deficiency was freshly drawn in blood collection tubes. Peripheral blood mononuclear cells (PBMCs) were isolated from blood by Biocoll density gradient centrifugation (Merck).

### Cell lines

Human Embryonic Kidney (HEK) cells and Chinese Hamster Ovary (CHO) cell lines were cultured in Dulbecco’s modified Eagle’s medium (DMEM) or Roswell Park Memorial Institute (RPMI) medium supplemented with 10% fetal calf serum (FCS), 1% sodium-pyruvate, 1% l-glutamine and 1% penicillin/streptomycin (10,000 U/mL). The human histiocytic lymphoma cell line U937 was grown in RPMI medium supplemented with 10% FCS, 1% l-glutamine and 1% penicillin/streptomycin (10,000 U/mL). The human glioblastoma cell line U87 was grown in DMEM supplemented with 10% FCS and 1% penicillin/streptomycin (10,000 U/mL). All cell lines were incubated at 37°C with 5% CO_2_, and passaging was routinely performed two to three times a week at 70% confluency (1:3–1:5). For adherent cell lines, trypsin was used to detach cells before splitting.

### Transfection of cell lines

HEK293 and CHO cells were transiently transfected with plasmids encoding human *ENPP1* (pCDNA3.1 vector), *ENPP4* (pCMV6 vector) and *ENPP5* (peGFP.N1 vector). For this, 5 µg plasmid *ENPP*-DNA with optional addition of 0.5 µg GFP (pCDNA3.1 vector) were mixed with 0.333 mg/mL Polyethylenimine (PEI, PolySciences) and added to the cells. 24 h after transfection, transfection efficiency was determined by flow cytometry, and cells were used for subsequent analyses.

### Immunization of alpacas

The immunization procedure was performed by Eurogentec (Belgium) according to approved local and national animal ethics protocols. Eukaryotic expression vectors for human ENPP1 (gene ID 5167) were cloned into pCMV-SPORT6. Two alpacas (SAL010 and SAL011) were immunized and boosted with ENPP1 expression vectors by ballistic immunization as described previously [27]. Each alpaca received four DNA immunizations with administration intervals of two weeks. Each dose consisted of 12 shots of plasmid-conjugated gold particles (1 µg of DNA conjugated onto 0.5 mg gold particles per shot) applied with a pressure setting at 600 psi into the skin. Three weeks after the final genetic immunization, a single boost with 2 × 10^7^ *ENPP1*-transfected HEK293 cells was given. At regular intervals, blood samples were collected to monitor the induction of the humoral immune response over time. For the isolation of mononuclear cells, blood was collected from these animals seven days after the fourth DNA immunization and seven days after the cell boost.

### Selection of ENPP1-specific heavy-chain antibodies

Mononuclear cells were isolated from 120 mL blood by Ficoll-Paque gradient centrifugation (GE HealthCare). RNA purified from these cells by innuPREP RNA Mini Kit (Analytik Jena) was subjected to cDNA synthesis with alpaca CH2-specific primers. The VHH-coding region was amplified by PCR with degenerated hcAb-specific primers. PCR products were purified from agarose gels, digested sequentially with SfiI and NotI (New England Biolabs) and cloned into the pHEN2 phagemid vector downstream of the PelB-leader peptide and upstream of the chimeric His6x-Myc epitope tag [27]. Transformation into XL1-Blue *E. coli* (Stratagene) yielded libraries with sizes of 1–3 × 10^6^ clones. The library was cloned into pCSE2.5 vector including rabbit Ig CH2 and CH3 domains to express hcAbs. 4 × 96 clones were expressed in HEK293-6E cells and supernatants were screened by immunofluorescence microscopy on *ENPP1*-transfected CHO cells. Hits were sequenced, grouped and individual hits were expressed in HEK293-6E cells and purified.

### Production and reformatting of nanobodies and heavy-chain antibodies

Representative VHHs of the identified families were expressed as hcAbs. The coding region of selected nanobodies was subcloned using NcoI and NotI into the pCSE2.5 vector upstream of either a myc-C-hexahistidin tag or the hinge and Fc-domains of rabbit IgG or human IgG1 harboring LALAPG mutations [28–30]. Recombinant Myc-His tagged nanobodies and rabbit IgG or human IgG1 hcAbs were expressed in transiently transfected HEK293-6E cells cultivated in serum-free medium. Six days post transfection, supernatants were harvested and cleared by centrifugation. Nanobodies and hcAbs in cell supernatants were quantified by SDS-PAGE and Coomassie staining relative to marker proteins of known quantities. Concentrations of purified nanobodies and hcAbs were measured using BCA assay. Myc-His tagged nanobodies were purified by immobilized metal affinity chromatography using Ni-NTA agarose (Sigma). Heavy-chain antibodies were purified by affinity chromatography using protein A-sepharose (GE HealthCare). CDR3 sequences of used hcAbs against ENPP1 are shown in Supplementary Table 1. Purified hcAbs were conjugated to Alexa Fluor 647 by NHS ester (Thermo Fisher Scientific).

### Evaluation of the sensitivity and specificity of produced heavy-chain antibodies

The binding specificities and sensitivities of the produced hcAbs were determined by flow cytometry using *ENPP1*-transfected HEK293 cells. HEK293 cells were incubated for 30 min with HEK293-6E cell supernatants containing rabbit IgG hcAbs (diluted 1:10 in PBS/0.1% BSA) or purified hcAbs. Following washing with PBS/0.1% BSA, bound antibodies were detected with PE-labeled donkey anti-rabbit IgG (Jackson ImmunoResearch) or AF647-labeled goat anti-rabbit IgG polyclonal antibody (Invitrogen). The cells were resuspended in PBS and analyzed by flow cytometry.

### Dissociation of ENPP1-specific heavy-chain antibodies from ENPP1-transfected HEK293 cells

For dissociation analyses, two separate groups of *ENPP1*-transfected HEK293 cells were either saturated with ENPP1-binding hcAbs or labeled with the dye eFluor 450 (eF450, Thermo Fisher Scientific) for 20 min at RT. Cell groups were washed separately four times and then combined at a 1:1 ratio. Directly after merging the cells (0 h) and after another incubation at RT for 2 h, cells were stained for bound antibodies with PE-conjugated donkey anti-rabbit IgG polyclonal antibody. The PE signal of the hcAbs on eF450^–^ and eF450^+^ cells was analyzed by flow cytometry. Dissociation rates of hcAbs were calculated as [(MFI PE on eF450^+^ cells/MFI PE on eF450^–^ cells) × 100] after 2 h.

### Detection of cell surface expression of ENPP1 by flow cytometry

hcAb SB66 was selected for the analysis of ENPP1 expression on the cell surface of human PBMCs and cell lines by flow cytometry. PBMCs were pre-incubated with IgG (5 min, RT) to block unspecific binding. For direct staining, cells were incubated for 30 min at RT with 10 µL of 1:10 pre-diluted AF647-conjugated SB66 rbFc or 1:30 pre-diluted SB66 hFc LALAPG hcAb. An AF647-conjugated rabbit IgG hcAb specific for murine ART2.2 was used as a negative control. For indirect staining, cells were stained with SB66 rbFc in combination with an anti-rabbit PE-labeled secondary antibody. Here, an irrelevant hcAb against L-10E in combination with the secondary antibody was used as negative control. PBMCs were co-stained with lineage markers to identify immune cell populations (see Supplementary Table 2 for a list with all antibodies used). Live/dead staining using an amine-reacting fluorescent live/dead dye (Alexa Fluor 750 succinimidyl ester, Thermo Fisher Scientific) was performed 10 min after staining with lineage markers. After washing, stained cells were measured at the flow cytometer (FACSCanto II, FACSCelesta or FACSymphony A1 (BD Biosciences)) and analyzed using the FlowJo software (BD Biosciences). PBMCs were identified in the forward scatter (FSC) versus side scatter (SSC) dot plot and staining with CD45. Dead cells were excluded from analysis based on the staining with live/dead dye. After exclusion of unwanted cell types by lineage marker expression, the relevant populations were identified as shown in the gating strategy of each figure. For each population of interest, we analyzed the median fluorescence intensity (MFI) of the ENPP1 staining and the frequency of ENPP1-positive cells, and compared it to the control staining.

### Statistics

Data representation and statistical analysis were performed using Prism 6 and Prism 10 (GraphPad). Detailed information regarding the used statistical tests are provided in the figure legends. For comparisons between two groups with paired data, paired t test (for parametric data) or Wilcoxon test (for nonparametric data) were used. For comparisons among multiple groups, repeated measures (RM) one-way ANOVA was used for paired, parametric data, and ordinary one-way ANOVA (for parametric data) or Kruskal–Wallis test (for nonparametric data) were used for unpaired data. For multiple comparisons, post hoc tests were performed as stated in the figure legends. Statistical significance is indicated as * p ≤ 0.05, ** p ≤ 0.01, *** *p* ≤ 0.001, **** p ≤ 0.0001. Non-significant differences are not annotated.

## Results

### Heavy-chain antibody SB66 recognizes human ENPP1 with high affinity and specificity

**Fig. 1:**
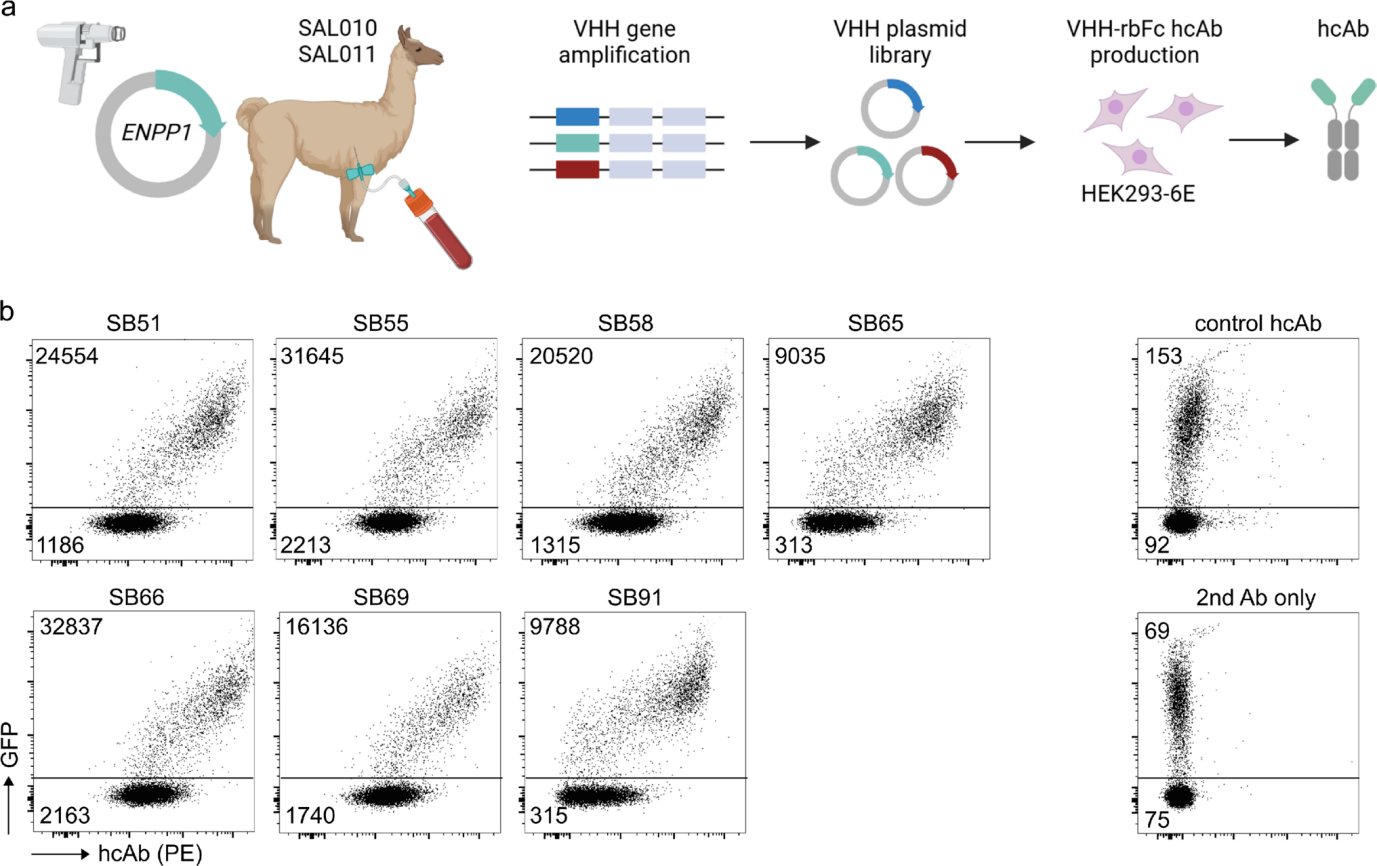
Selection of human ENPP1-binding heavy-chain antibodies. **a** Schematic representation of the generation of ENPP1-specific hcAbs. **b** Flow cytometric analysis of the binding of generated hcAbs (in HEK293-6E cell supernatant) to *ENPP1*-transfected HEK293 cells. The binding of hcAbs was detected with PE-labeled donkey anti-rabbit IgG. Median fluorescence intensity (MFI) values of the PE-signal from GFP^+^ and GFP^–^ populations are indicated in the plot. Control stainings were performed using an irrelevant L-10E rbFc hcAb and the secondary PE-labeled donkey anti-rabbit IgG antibody or the secondary antibody alone

Measuring ENPP1 protein expression on a single-cell level in humans is hampered by the lack of monoclonal antibodies suitable for flow cytometry. Here, we set out to generate heavy-chain antibodies (hcAbs) targeting ENPP1. For this, we immunized two alpacas (SAL010 and SAL011) with human ENPP1, as described previously [27]. After isolation of peripheral blood mononuclear cells (PBMCs) from the immunized alpacas, we amplified the coding region of the single antigen-binding variable domain (nanobody/VHH) and cloned it N-terminally to a rabbit Fc portion (rbFc) to reconstitute a heavy-chain antibody format. The resulting hcAbs were expressed in HEK293-6E cells, screened on *ENPP1*-transfected cells, and the ENPP1-binders were sequenced, grouped, expressed in HEK293-6E cells, and purified (Fig. 1a). To evaluate the staining intensity of the hcAb clones, we used HEK293 cells co-transfected with green fluorescent protein (GFP) and human *ENPP1*. The hcAbs SB51, SB55, SB58, SB65, SB66, SB69 and SB91 showed a 10 to 30-fold higher signal on GFP-positive *ENPP1*-transfected HEK293 cells compared to non-transfected cells (Fig. 1b), verifying that the hcAb clones bind human ENPP1 on the cell surface.

**Fig. 2:**
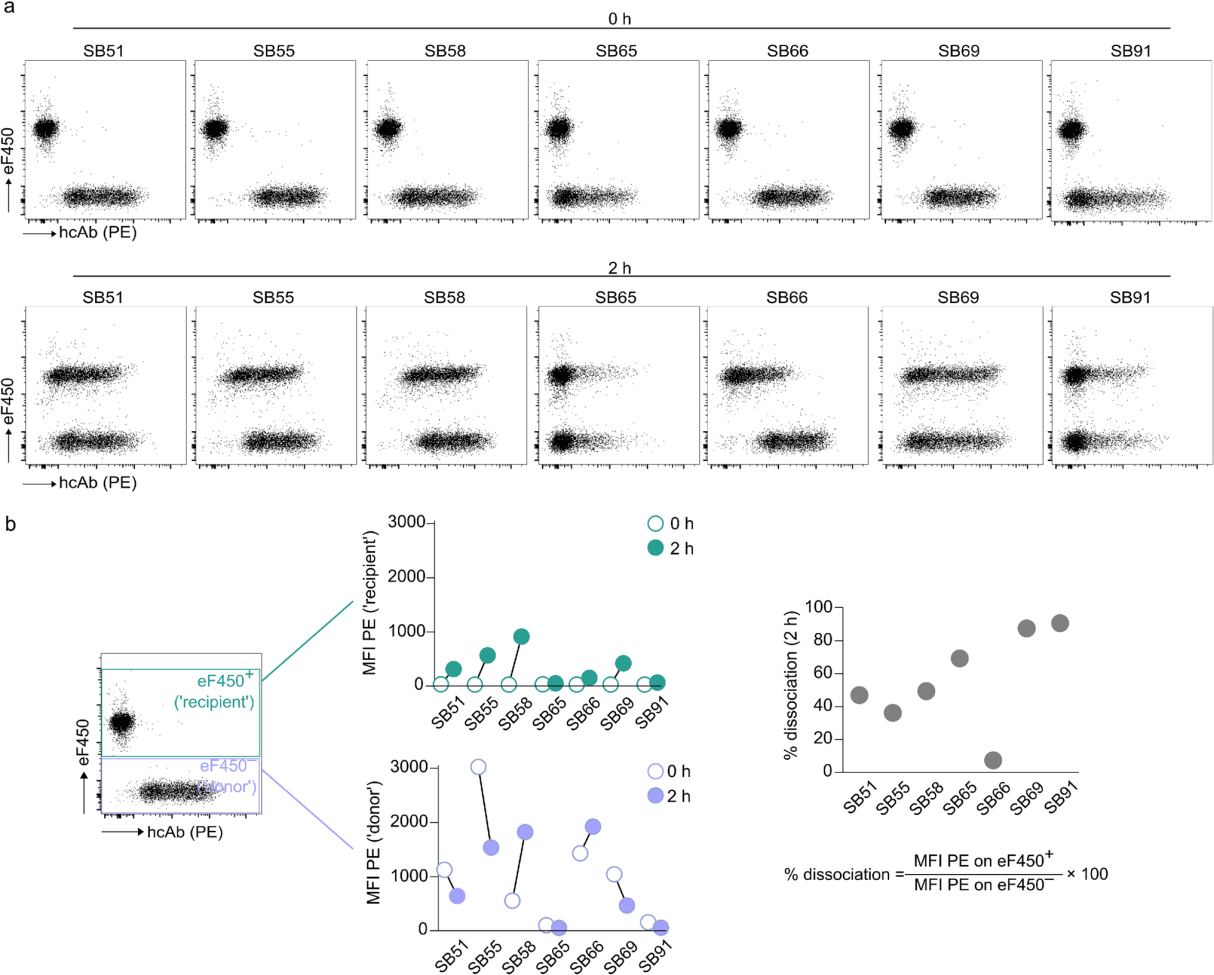
ENPP1-binding hcAb SB66 exhibits a low dissociation from ENPP1.** a** *ENPP1*-transfected HEK293 cells were either saturated with ENPP1-binding hcAbs (‘donor cells’) or labeled with eFluor 450 (eF450) (‘recipient cells’). Cells from the two groups were mixed 1:1 and stained immediately (0 h) and after 2 h with PE-labeled donkey anti-rabbit IgG and analyzed by flow cytometry. **b** The MFI of the secondary antibody (PE) was compared at 0 h and 2 h for both ‘eF450^–^ donor cells’ and ‘eF450^+^ recipient cells’. Dissociation rates of hcAbs were calculated as [(MFI PE on eF450^+^ cells/MFI PE on eF450^–^ cells) × 100] at the 2 h time point

In a subsequent validation step, we examined the dissociation kinetics of the hcAbs. For this, we mixed ‘donor cells’ (*ENPP1*-transfected HEK293 cells that were saturated with ENPP1-binding hcAbs) in a 1:1 ratio with eFluor 450 (eF450)-labeled ‘recipient cells’ (*ENPP1*-transfected HEK293 cells that did not get in contact with hcAbs). After two hours of co-incubation, we assessed the dissociation of the hcAbs from the ‘donor cells’ (eF450^–^) and subsequent binding to the ‘recipient cells’ (eF450^+^) by staining with a PE-labeled secondary antibody against rbFc (Fig. 2a, b). The ratio of the median fluorescence intensity (MFI) between the two groups indicated that clone SB66 showed the lowest dissociation (< 5%) of all hcAbs (Fig. 2b). Altogether, we conclude that the hcAb clone SB66 has a high affinity and a low dissociation for binding human ENPP1 on the cell membrane. Therefore, clone SB66 was chosen for all upcoming experiments.

**Fig. 3:**
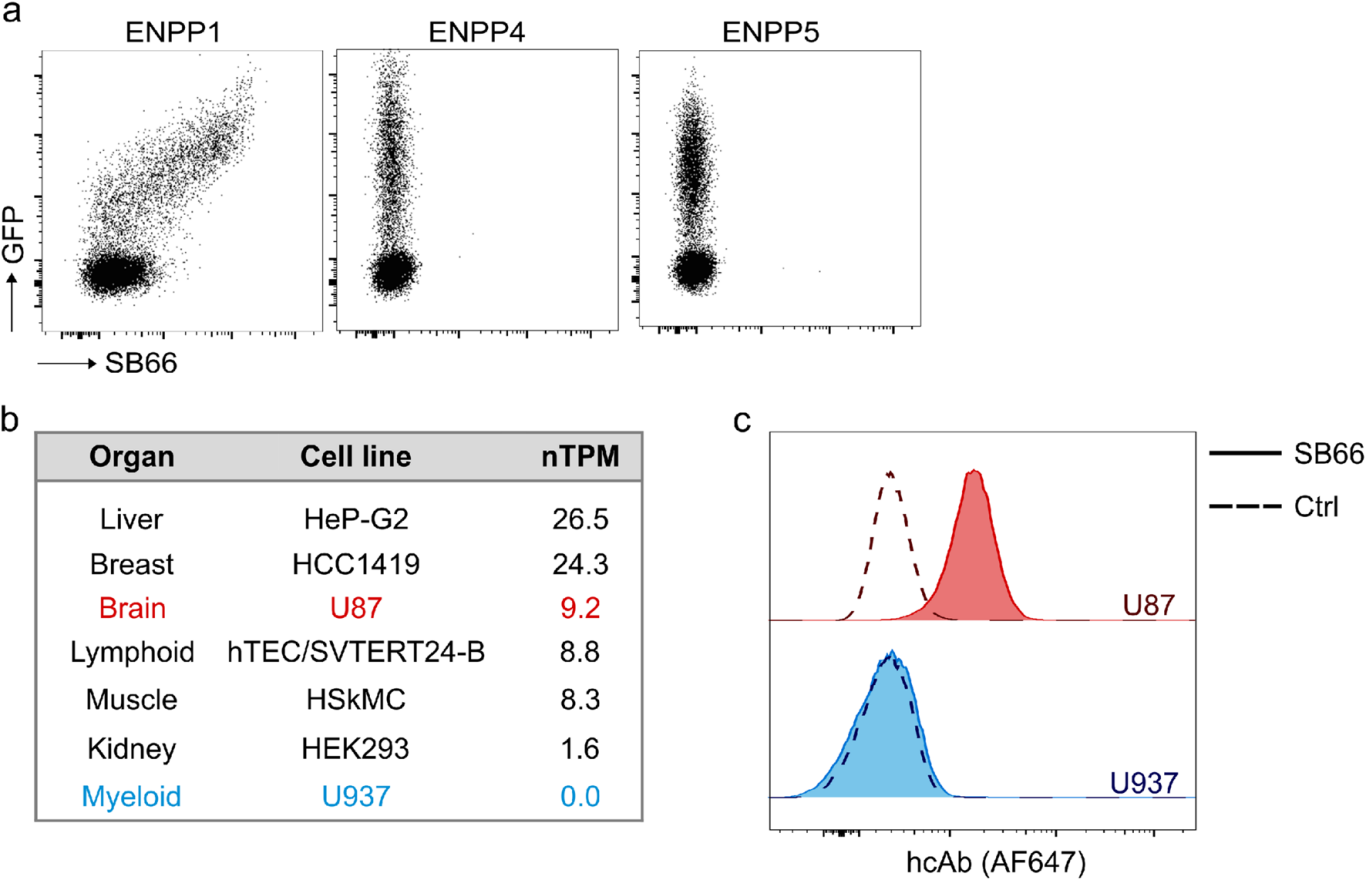
ENPP1-binding hcAb SB66 specifically detects ENPP1.** a** Flow cytometric analysis of the binding of hcAb SB66 to *ENPP1-*, *ENPP4-* and *ENPP5*-transfected HEK293 cells. The binding of SB66 was detected with AF647-labeled goat anti-rabbit IgG. **b** *ENPP1* RNA expression in selected cell lines, according to data from the Human Protein Atlas (https://www.proteinatlas.org/). **c** Flow cytometric analysis of ENPP1 expression on the human glioblastoma cell line U87 and the pro-monocytic myeloid leukemia cell line U937 using SB66 hFc LALAPG-AF647. Control staining was performed with anti-mouse ART2.2 rbFc-AF647

The next step aimed to evaluate the specificity of SB66 for ENPP1 against other ENPP family members potentially expressed on human immune cells. For this, we transfected HEK293 cells with *ENPP4* or *ENPP5*. Staining with SB66 only showed a signal for *ENPP1*-transfected cells, but not for HEK293 cells transfected with *ENPP4* or *ENPP5* (Fig. 3a). hcAbs are soluble and compact in size, have a high stability and their Fc region can be easily reformatted. To assess whether we can use the anti-ENPP1 hcAb SB66 in different formats for flow cytometry, we purified SB66 from HEK293 cell culture supernatants, and used SB66 rbFc either directly conjugated to the fluorochrome AF647, or in an indirect staining approach using unconjugated SB66 rbFc followed by PE-labeled donkey anti-rabbit IgG. Additionally, we utilized a ‘silent’ human IgG1 Fc moiety with a LALAPG mutation to minimize binding to Fcγ receptors [30]. When using *ENPP1*-transfected HEK293 cells, we saw a good staining of ENPP1 with all above mentioned approaches, although the background signal was slightly higher in the secondary staining approach (Supplementary Fig. 1). Next, we wanted to test SB66 on cell lines that endogenously express ENPP1. In agreement with RNA data of the Human Protein Atlas (Fig. 3b) [31], we found ENPP1 protein expression on the glioblastoma cell line U87, but not on the pro-monocytic myeloid leukemia cell line U937 (Fig. 3b, c). Our results demonstrate that we can reliably use the hcAb SB66 in various formats to assess ENPP1 not only on transfected cells, but also on cell lines with endogenous ENPP1 expression.

### *ENPP1 is highly expressed on CD141*^+^*conventional DCs and CD56*^*bright*^* NK cells*

**Fig. 4:**
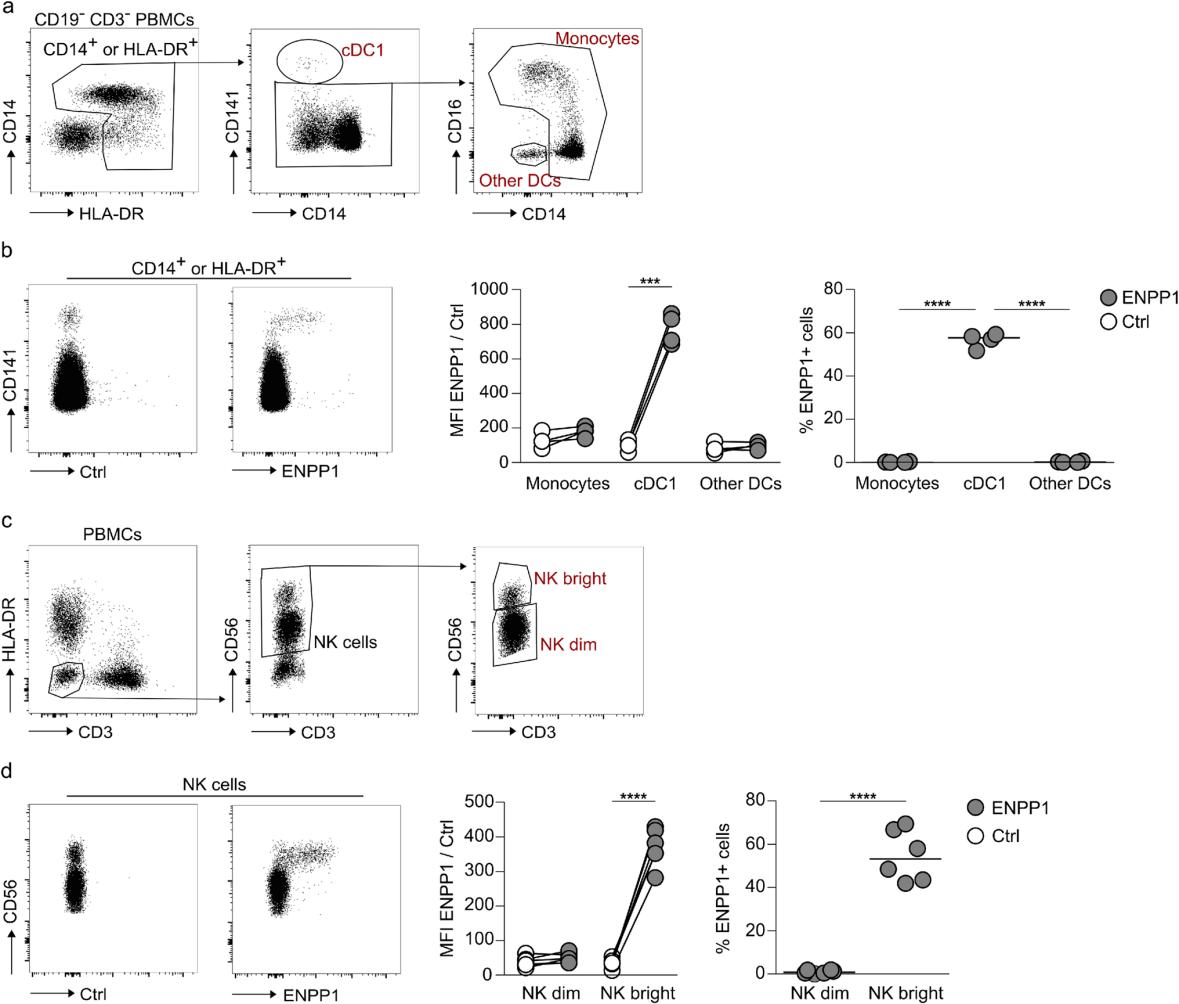
ENPP1 is highly expressed on cDC1 and CD56^bright^ NK cells.** a** Gating strategy to identify monocytes and DC subsets. **b** MFI and frequency (%) of cells expressing ENPP1 in monocytes and DC subsets (n = 4, median marked as line). ENPP1 staining was performed with SB66 rbFc-AF647, and anti-mouse ART2.2 rbFc-AF647 was used for control staining. **c** Gating strategy to identify NK cell subsets. **d** MFI and frequency of NK cells expressing ENPP1 (n = 6, median marked as line). ENPP1 staining was performed with SB66 hFc LALAPG-AF647, and anti-mouse ART2.2 rbFc-AF647 was used for control staining. **b**, **d** Paired t test was used to compare the MFI of cells stained with the ENPP1-specific and the control hcAb. RM one-way ANOVA with Tukey’s multiple comparisons test (**b**) or paired t test (**d**) was used to compare the frequency of ENPP1-positive cells among different cell subsets (*p ≤ 0.05, **p ≤ 0.01, ***p ≤ 0.001, ****p ≤ 0.0001)

Transcriptomic data, such as the Human Cell Atlas [24] or the Human Protein Atlas [31] provide valuable insights into *ENPP1* RNA expression. However, there are no data on surface protein expression of ENPP1 in human immune cells. To address this gap, we employed multicolor flow cytometry and combinations of lineage markers to assess the frequency and expression levels of ENPP1 on peripheral blood cell subpopulations. As negative control, we used a hcAb against murine ART2.2, a molecule lacking a human orthologue, labeled with the same fluorophore. Within the myeloid cell compartment, we identified monocytes and DCs (gating strategy in Fig. 4a). With around 55%, we found the highest expression of ENPP1 in CD141^high^ conventional DCs (cDC1), while the percentages of other DCs and monocytes expressing ENPP1 were below 0.5% (Fig. 4b). The next innate cell subset analyzed were NK cells, identified as CD3^─^ HLA-DR^─^ CD56^+^ (gating strategy in Fig. 4c). ENPP1 was exclusively expressed in NK cells expressing high levels of CD56 (hereinafter named NK bright). This immature population of NK cells showed a frequency of around 50% ENPP1-positive cells (Fig. 4d). In contrast, less than 1% of mature CD56^dim^ NK cells (referred to as NK dim) are ENPP1-positive. In summary, these data show that we can reliably detect ENPP1 on the surface of primary human immune cells using hcAb SB66, and that, within innate cells, cDC1s and CD56^bright^ NK cells express ENPP1.

### ENPP1 is not expressed on B lymphocytes or circulating plasmablasts

**Fig. 5:**
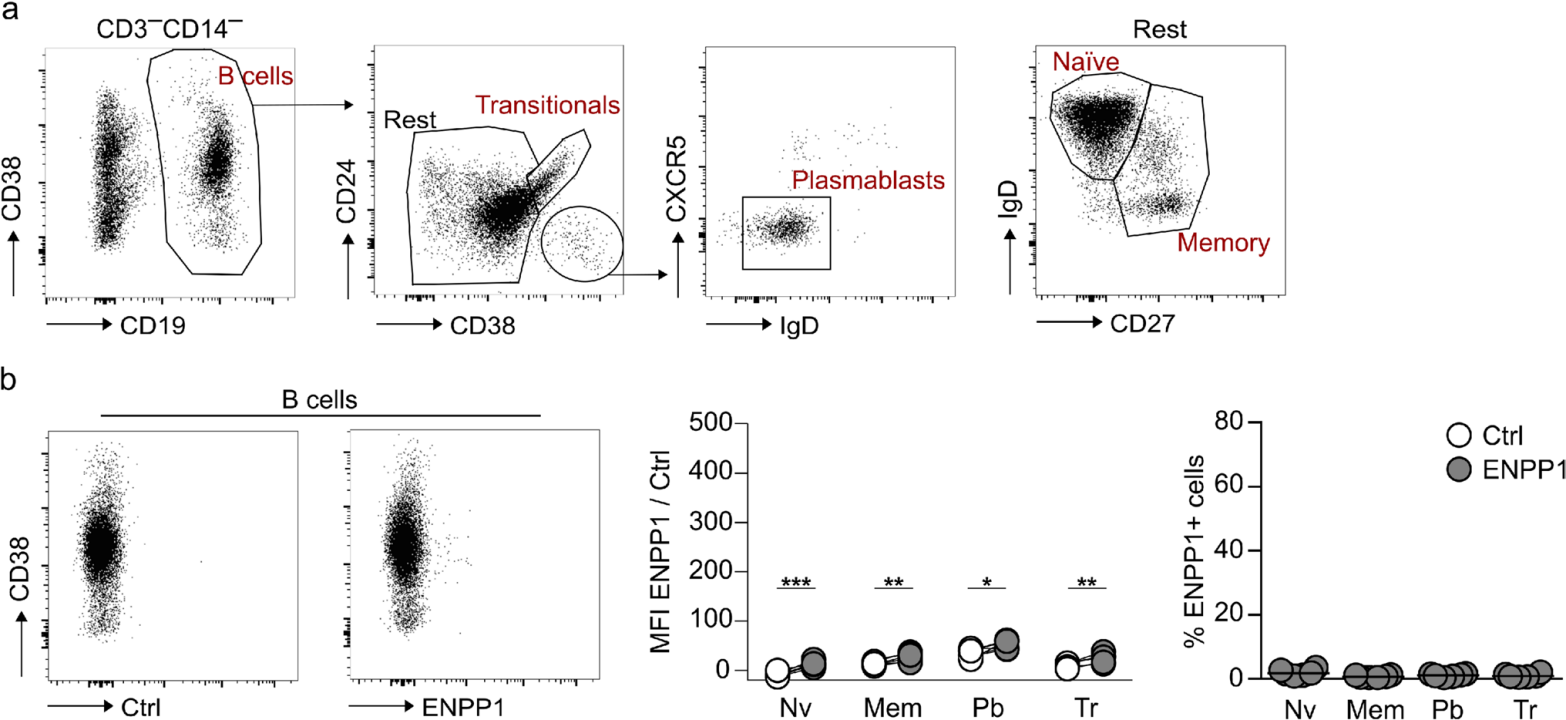
ENPP1 is not expressed on human peripheral blood B cells and plasmablasts.** a** Gating strategy to determine different B cell populations. **b** MFI and frequency of ENPP1 cell surface expression on B cell subsets (n = 6, median marked as line). ENPP1 staining was performed with SB66 hFc LALAPG-AF647, and anti-mouse ART2.2 rbFc-AF647 was used for control staining. Paired t test was used to compare the MFI of cells stained with the ENPP1-specific and the control hcAb, RM one-way ANOVA with Tukey's multiple comparisons test was used to compare the frequencies of ENPP1-positive cells among different B cell subpopulations (*p ≤ 0.05, **p ≤ 0.01, ***p ≤ 0.001, ****p ≤ 0.0001). Nv, Naïve; Mem, Memory; Pb, Plasmablasts; Tr, Transitional

ENPP1 was initially characterized as a plasma cell-defining molecule in mice, and it is therefore also known as plasma cell antigen 1 (PC-1) [5]. Two previous reports show ENPP1 expression in human peripheral blood and bone marrow plasma cells [32, 33], while gene expression datasets such as Human Cell Atlas [24] and the Human Protein Atlas [31] show low ENPP1 expression on B cells, exclusively in the naïve subset. Using a combination of markers for B cell differentiation, we identified transitional, naïve and memory B cells, as well as plasmablasts (gating strategy in Fig. 5a). ENPP1 expression was measured using the hcAb SB66 hFc LALAPG-AF647 to avoid binding to Fcγ receptors, and compared to control staining with anti-mouse ART2.2 rbFc-AF647. Even though the difference in the MFI between the ENPP1- and control staining is statistically significant, the level of expression is extremely low, and the frequency of ENPP1-positive cells is practically negligible as only 1–2% of all analyzed B cell populations express ENPP1 (Fig. 5b). We repeated the staining of ENPP1 on plasmablasts using unlabeled SB66 rbFc in combination with a secondary antibody to enhance a potential positive signal. However, also with this approach we could not detect ENPP1 on human plasmablasts (data not shown). We conclude that circulating human B cells, including plasmablasts, do not express ENPP1, underscoring striking differences in ENPP1 expression in mice and humans.

### MAIT cells are the only T cell subset constitutively expressing ENPP1

**Fig. 6:**
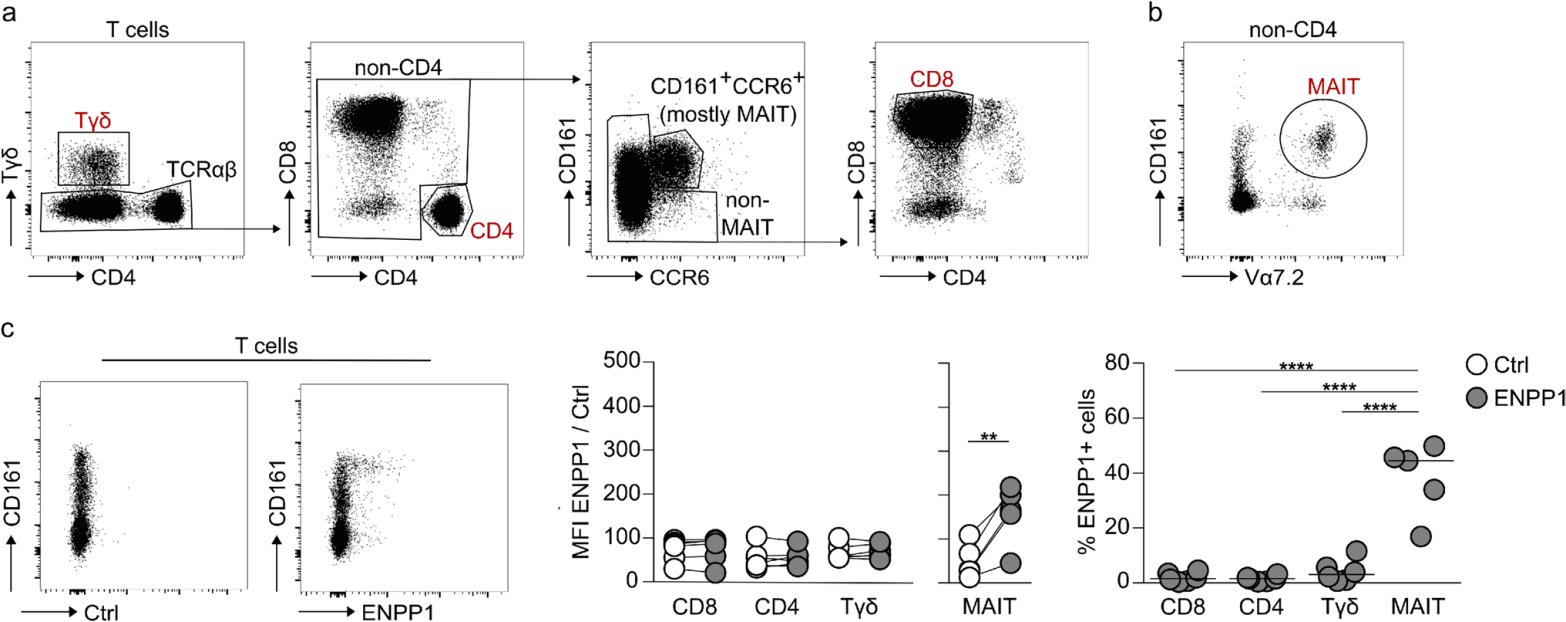
ENPP1 is expressed on MAIT cells. **a**, **b** Gating strategies to identify different T cell populations, including conventional CD4 and CD8 T cells, γδ T cells (**a**) and MAIT cells (**b**). **c** MFI and frequency of ENPP1 cell surface expression on T cell subpopulations (n = 5–6, median marked as line). ENPP1 staining was performed with SB66 rbFc, in combination with a secondary PE-labeled donkey anti-rabbit IgG antibody. Control staining was performed using L-10E rbFc and the secondary PE-labeled donkey anti-rabbit IgG antibody. Wilcoxon test (CD4, CD8, Tγδ) or paired t test (MAIT) was used to compare the MFI of cells stained with the ENPP1-specific and the control hcAb. Ordinary one-way ANOVA with Tukey’s multiple comparisons test was used to compare the expression of ENPP1 among different T cell subsets (*p ≤ 0.05, **p ≤ 0.01, ***p ≤ 0.001, ****p ≤ 0.0001). MAIT, Mucosal-associated invariant T cells

We next sought to investigate the expression of ENPP1 on T cells, namely conventional CD4- and CD8-positive T lymphocytes, innate-like γδ T cells and mucosal-associated invariant T (MAIT) cells (gating strategy in Fig. 6a, b). ENPP1 is not expressed on CD4-expressing T cells, which includes conventional CD4 T cells and regulatory T cells (Treg) (Fig. 6c). Also γδ T cells do not express ENPP1. In the CD4-negative compartment (non-CD4), we did not find ENPP1 on conventional CD8 T cells, but on CD161^+^CCR6^+^ cells, a subset containing mostly MAIT cells. Because the labeling with CCR6 and CD161 is not completely specific for MAIT cells, we used a second gating strategy that considers the invariant T cell receptor alpha chain 7.2 (Vα7.2) together with CD161 to unmistakably identify this cell type (Fig. 6b). Indeed, we confirmed that ENPP1 is expressed in 20 to 50% of MAIT cells (Fig. 6c).

**Fig. 7:**
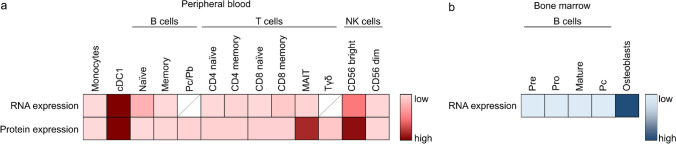
Correlation between RNA and cell surface protein expression of ENPP1 for different immune cell populations. **a** *ENPP1* RNA expression data (normalized expression values) were obtained from the Human Cell Atlas (https://data.humancellatlas.org/) [24]. The median frequency of ENPP1 protein expression was obtained from the data presented in this paper (n = 4–6). Each row was compared and colorized individually. **b** *ENPP1* RNA expression data (normalized expression values) were obtained from a human bone marrow dataset (https://cscb.research.chop.edu/vitessce/demo-seq/build/) [34]. cDC1, Conventional dendritic cell type 1; MAIT, Mucosal-associated invariant T cells; NK, Natural killer; Pb, Plasmablast; Pc, Plasma cell

In summary, we find that ENPP1 is expressed at highest levels in cDC1, followed by CD56^bright^ NK cells, and MAIT cells. The only existing data on ENPP1 expression on several immune cell populations before our analysis could be extracted from RNA datasets. Therefore, we compared the RNA sequencing data from the Human Cell Atlas [24] with the protein data that we obtained by flow cytometry analysis (Fig. 7a). Also in the RNA dataset, cDC1 and CD56^bright^ NK cells show the highest expression of *ENPP1*. However, MAIT cells show similar levels of *ENPP1* RNA expression as other T cell subpopulations, which does not correspond to the protein expression that we describe (Fig. 6c). This difference may be due to the selection of markers for sorting this cell type for the RNA analysis. In the Human Cell Atlas dataset, only CD8^+^ Vα7.2^+^ CD161^+^ MAIT cells are considered [24], while we also included CD8-negative MAIT cells. Finally, the analysis of the B cell compartment brought the unexpected finding of no expression of ENPP1 in human circulating antibody-secreting cells (plasmablasts), even though ENPP1 was first described in murine plasma cells [5, 6]. Because long-lived plasma cells reside in the bone marrow, we checked for *ENPP1* expression in an existing dataset of human adult bone marrow cells [34] (Fig. 7b). The data confirms that ENPP1 is not expressed at any stage of B cell maturation in human bone marrow, including plasma cells. As expected, osteoblasts in the same bone marrow samples clearly express *ENPP1*, serving as a positive control.

### hcAb SB66 constitutes a quick diagnostic tool for detecting ENPP1 deficiency in patients with bone fragility

**Fig. 8:**
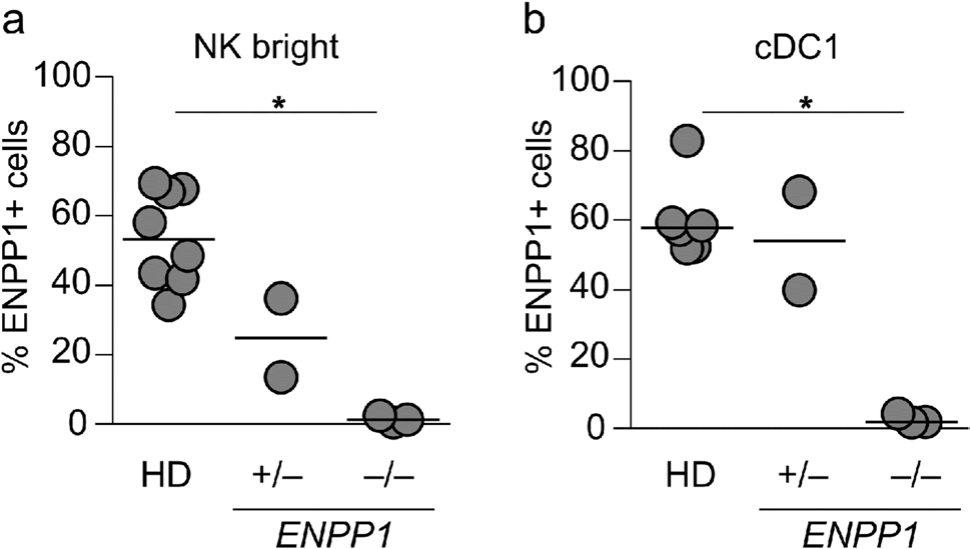
ENPP1 surface expression is not detectable in patients with *ENPP1* deficiency. **a**, **b** ENPP1 expression in healthy donors (HD, n = 8, median marked as line) and patients with a monoallelic (*ENPP1*^+/−^, n = 2) or biallelic (*ENPP1*^–/–^, n = 3) *ENPP1* deficiency was measured by flow cytometry. The frequency of ENPP1 is shown for NK bright (**a**) and cDC1 (**b**) cells. ENPP1 staining was performed using SB66 hFc LALAPG-AF647 or SB66 rbFc-AF647. Kruskal–Wallis test with Dunn’s multiple comparisons test was used to compare the expression of ENPP1 among different donors (*p ≤ 0.05, **p ≤ 0.01, ***p ≤ 0.001, ****p ≤ 0.0001)

Osteoblasts express high levels of ENPP1 (Fig. 7b). ENPP1 metabolizes ATP to generate pyrophosphates, necessary for bone formation. Patients with a deficiency in *ENPP1* suffer from arterial calcification early and from ARHR2 later in life, characterized by low bone mineral density due to the lack of pyrophosphates. We compared ENPP1 expression in healthy donors and in patients with either monoallelic or biallelic mutations in *ENPP1*. Our analysis reveals a complete absence of ENPP1 on the cell surface of NK bright and cDC1 cells in individuals with both alleles affected (*ENPP1*^−/−^), while individuals with only one affected allele (*ENPP1*^+/−^) showed only a partial reduction or no reduction at all in the frequency of NK bright and cDC1 cells expressing ENPP1 compared to healthy donors (HD) (Fig. 8a, b). These findings confirm the specificity of hcAb SB66 in a setting of human deficiency, and underscore the utility of this reagent as a reliable and quick diagnostic tool for the detection of *ENPP1* deficiency in easily accessible peripheral blood.

## Discussion

ENPP1 is a cell surface pyrophosphatase/phosphodiesterase that hydrolyzes different extracellular substrates, including the cGAS/STING pathway second messenger 2′3′-cGAMP, and the P2 receptor agonist ATP. Here, we generated and characterized human ENPP1-specific hcAbs, and used them to measure the cell surface expression of ENPP1 on a broad variety of immune cells by multicolor flow cytometry. We show that ENPP1 is expressed on very specific immune cell subsets, namely cDC1, CD56^bright^ NK cells and MAIT cells. Further, by comparative analysis, we establish the parallels with existing RNA expression data, and pinpoint striking differences in ENPP1 expression between mouse and human B cells.

In the last years, ENPP1 has become an attractive target for immunotherapy because of its expression on cancer cells and its capacity to modulate the cGAS/STING pathway. However, due to the lack of commercially available antibodies suitable for flow cytometry, not much is known on the expression and function of ENPP1 on human immune cells. We found that the expression of ENPP1 is highly cell-specific, and the three ENPP1-expressing immune cell types, cDC1s, CD56^bright^ NK cells and MAIT cells, are paradoxically unrelated in terms of ontogeny and function. A possibility is that ENPP1 has a distinct function on each of the cell types, depending on the purinergic substrates recognized by cell surface receptors. For instance, NK and MAIT cells express the ATP receptor P2X7 at higher levels than any other lymphocyte population [35]. Engagement of P2 receptors induces cell activation and may result in cell death. The expression of ENPP1 in the same cell could be a way to reduce the availability of ATP for P2 receptors, preventing activation and death. CD56^dim^ NK cells are the majority of NK cells in peripheral blood, while CD56^bright^ NK cells constitute the dominant NK subset in secondary lymphoid tissues, where the concentration of extracellular ATP is higher. NK cells only minimally express CD39, another ATP-degrading enzyme. Therefore, it is plausible that ENPP1 protects the more immature CD56^bright^ NK cells from early activation and death in lymph nodes and spleen.

cGAMP is produced by the cytoplasmic sensor cGAS in response to the presence of dsDNA associated with pathogens or cellular damage. cGAMP activates STING to induce type I interferons and pro-inflammatory cytokines essential for anti-viral and anti-tumor responses. Enabled by exporters and importers, cGAMP can travel to neighboring cells [36, 37], where it activates STING independently of DNA sensing by cGAS [20, 38]. While residing in the extracellular milieu, cGAMP is susceptible of degradation by ENPP1 expressed on either cGAMP ‘donor’ or ‘recipient’ cells, or on extracellular vesicles [39, 40]. Interestingly, the ENPP1-expressing population cDC1 are essential for STING-mediated rejection of established and metastatic murine tumors [41], and blocking ENPP1 in these cells could enhance the STING pathway and type I interferon response, contributing to anti-tumor immunity.

The expression of purinergic enzymes in immune cells of different species is discordant [42]. CD73, for instance, is expressed on murine macrophages and regulatory T cells (Tregs), while in humans it is expressed on most B cells, but on very few Tregs or monocytes. ENPP1 was initially identified as a surface protein on plasma cells (and consequently named PC-1). Indeed, RNA data show that mice have the highest expression of *ENPP1* on plasma cells [43]. In humans, *ENPP1* expression is very low in the B cell compartment, and predominantly in naïve B cells [24, 31]. Previous studies on human PBMCs report expression of ENPP1 on plasma cells and an upregulation of ENPP1 on B cells after activation [32]. Our data show a small proportion (less than 2%) of naïve B cells expressing ENPP1, and even fewer ENPP1-positive cells in other B cell populations, including plasmablasts. The function of ENPP1 on plasma cells in mice is controversial. On the one hand, the enzyme appears to be essential for long-term survival and antibody production [33]. On the other hand, it has been reported that plasma cells depend on ATP-dependent P2X4 signaling [44], which contradicts with the ATP-degrading function of ENPP1. Further analysis of ENPP1 expression in inflamed organs will be necessary to ascertain the function of this ectonucleotidase on B cells.

ENPP1 is overexpressed in many cancers, and ENPP1 inhibitors or anti-ENPP1-drug conjugates for anti-cancer therapy are intensively discussed [23, 45]. Notably, a variable heavy (VH) single-domain antibody has shown promise as potential biological inhibitor, with the capability of bispecific fusion with a PD-L1 inhibitor [46]. Administration of an antibody against ENPP1 also rescues post-infarct metabolic defects in a humanized mouse model of myocardial infarction [47]. For patients with *ENPP1* deficiency, ERT is already under investigation in a phase 1/2 study (NCT04686175, ClinicalTrials.gov). To avoid off-target effects, it is critical to understand the function of ENPP1 on the different cell types, including immune cells. Systemic ENPP1 inhibition could have adverse side effects on soft tissue calcification and bone mineralization, and low ENPP1 activity would result in accumulation of extracellular cGAMP, leading to activation of the STING pathway and an exacerbated type I interferon response [48]. On the other side, ENPP1 substitution might affect immune function by reducing type I interferon responses.

hcAbs are easier to modify into different formats than conventional antibodies. The produced ENPP1-binding hcAbs could be modified for cell-specific targeting of tumor cells using bispecific nanobodies, which are expected to have less secondary effects than systemic ENPP1 inhibition. Additionally, our designed hcAb offers a novel diagnostic approach to detect ENPP1 as biomarker in tumors, and to screen enzyme levels in *ENPP1* deficiency and during ERT. Further studies could determine if there is a correlation between ENPP1 surface expression on immune cells and the severity of *ENPP1* deficiency.

In summary, we have generated hcAbs that can be used for the sensitive detection of ENPP1 on the cell surface, and allowed the identification of very distinct immune cell populations expressing ENPP1, namely CD56^bright^ NK cells, cDC1, and MAIT cells. This information can be used to further elaborate on the function of ENPP1 on these cells, as well as to evaluate and prevent potential systemic effects of ENPP1 inhibition, currently discussed as anti-cancer therapy.

## Supplementary Information

Below is the link to the electronic supplementary material.

Supplementary file1 (DOCX 9029 KB)

## Data Availability

The authors declare that the data supporting the findings of this study are available within the paper and its supplementary information files. Data from publicly available sources shown in this paper can be obtained from the Human Protein Atlas (https://www.proteinatlas.org/) [31], the Human Cell Atlas (https://data.humancellatlas.org/) [24] or a human bone marrow dataset (https://cscb.research.chop.edu/vitessce/demo-seq/build/) [34].
