# Supplementary material for: ENPP1/CD203a-targeting heavy-chain antibody reveals cell-specific expression on human immune cells
Source: Cell Mol Life Sci. 2024 Dec 18;82(1):6. doi: 10.1007/s00018-024-05539-y (PMC11655721; doi:10.1007/s00018-024-05539-y)
Supplement: Supplementary file 1 — Supplementary file1 (DOCX 9029 KB) [file 18_2024_5539_MOESM1_ESM.docx]

# Supplementary information

**Supplementary Table 1.** CDR3 sequences of used hcAbs against ENPP1.

| **Clone** | **CDR3 sequence** | **Species reactivity** |
| --- | --- | --- |
| **SB51** | EWTWVDGSKATMLGRSTADY | h |
| **SB55** | APDYSGIYCLTSSYRYLDL | h + m |
| **SB58** | APEYSGIYCLTSSYRYLDL | h + m |
| **SB65** | TAPDFGGRVSGEATYQY | h + m |
| **SB66** | EVRECRTRDEYWLYDL | h |
| **SB69** | VSPRYGSPDCAYDF | h + m |
| **SB91** | EVREWCRTRDEFWLYDL | h |

h = human, m = mouse

**Supplementary Table 2. List of antibodies/hcAbs used in this study.** All used antibodies/hcAbs are directed against human antigens if not indicated otherwise.

|  | **Specificity** | **Fluorochrome** | **Clone** | **Company** |
| --- | --- | --- | --- | --- |
| **Antibodies** | CD3 | Brilliant Violet 650 | OKT3 | BioLegend |
|  | CD3 | Alexa Fluor 700 | OKT3 | BioLegend |
|  | CD4 | PE/Cyanine7 | RPA-T4 | BioLegend |
|  | CD4 | Alexa Fluor 488 | RPA-T4 | BioLegend |
|  | CD8 | Brilliant Violet 510 | RPA-T8 | BioLegend |
|  | CD8a | Brilliant Violet 785 | RPA-T8 | BioLegend |
|  | CD14 | Alexa Fluor 700 | M5E2 | BioLegend |
|  | CD16 | Brilliant Violet 605 | 3G8 | BioLegend |
|  | CD19 | PE/Cyanine7 | HIB19 | BioLegend |
|  | CD24 | PerCP/Cyanine5.5 | ML5 | BioLegend |
|  | CD27 | Brilliant Violet 650 | O323 | BioLegend |
|  | CD38 | Alexa Fluor 488 | HIT2 | BioLegend |
|  | CD45 | Brilliant Violet 510 | HI30 | BioLegend |
|  | CD45 | Brilliant Violet 785 | 2D1 | BioLegend |
|  | CD45 | Alexa Fluor 488 | HI30 | BioLegend |
|  | CD56 | Brilliant Violet 421 | HCD56 | BioLegend |
|  | CD141 | PE | M80 | BioLegend |
|  | CD161 | Brilliant Violet 421 | HP-3G10 | BioLegend |
|  | CD161 | Brilliant Violet 605 | HP-3G10 | BioLegend |
|  | CD185 (CXCR5) | PE/Dazzle 594 | J252D4 | BioLegend |
|  | CD196 (CCR6) | PerCP/Cyanine5.5 | G034E3 | BioLegend |
|  | TCR γδ | Brilliant Violet 605 | 11F2 | BD |
|  | TCR γδ | PE/Cyanine7 | 11F2 | BioLegend |
|  | TCR Vα7.2 | APC | 3C10 | BioLegend |
|  | HLA-DR | FITC | G46-6 | BD |
|  | HLA-DR | Brilliant Violet 785 | L243 | BioLegend |
|  | HLA-DR | Brilliant Violet 711 | L243 | BioLegend |
|  | IgD | Brilliant Violet 510 | IA6-2 | BioLegend |
|  |  |  |  |  |
| **Heavy-chain antibodies** | SB51 rbFc | - | SB51 |  |
|  | SB55 rbFc | - | SB55 |  |
|  | SB58 rbFc | - | SB58 |  |
|  | SB65 rbFc | - | SB65 |  |
|  | SB66 rbFc | - | SB66 |  |
|  | SB66 rbFc | Alexa Fluor 647 | SB66 |  |
|  | SB66 hFc LALAPG | Alexa Fluor 647 | SB66 |  |
|  | SB69 rbFc | - | SB69 |  |
|  | SB91 rbFc | - | SB91 |  |
|  | Anti-mouse ART2.2 rbFc | Alexa Fluor 647 | s-14 |  |
|  | Anti-Toxin A L-10E rbFc | - | L-10E |  |
|  |  |  |  |  |
| **Secondary** | Donkey anti-rabbit IgG (H+L) | PE |  | Jackson |
| **antibodies** | Goat anti-rabbit IgG (H+L) | Alexa Fluor 647 |  | Invitrogen |
|  |  |  |  |  |


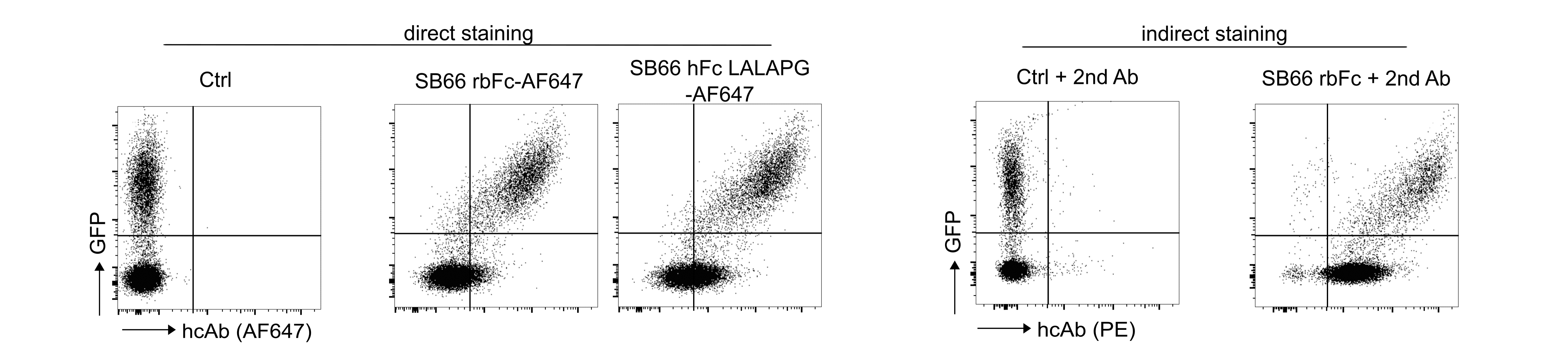
**Supplementary Figure 1. Heavy-chain antibody SB66 can be used in different formats to detect human ENPP1 on the cell surface.** Flow cytometric analysis of ENPP1 expression on HEK293 cells co-transfected with GFP and human ENPP1. Staining was performed with SB66 rbFc-AF647, SB66 hFc LALAPG-AF647 and SB66 rbFc, secondary stained with PE-labeled donkey anti-rabbit IgG. Control staining was performed with ART2.2 rbFc-AF647 or L-10E rbFc in combination with PE-labeled donkey anti-rabbit IgG.
